# The optimal substrate choice for effective biosensors based on THz metasurfaces

**DOI:** 10.1038/s41598-025-15669-3

**Published:** 2025-08-13

**Authors:** Pradeep Tiwari, Deepak Kala, Maciej Sakowicz

**Affiliations:** Institute of High Pressure Physics PAS, CENTERA Laboratories, Sokolowska 29/37, 01-142 Warsaw, Poland

**Keywords:** Metasurface, Terahertz, Simulations, Substrate, Biosensing, BSA, Applied physics, Applied optics

## Abstract

This article explores the impact of substrate choice on the sensitivity of sensors that utilize metallic terahertz metasurfaces as the actuating element. While terahertz metasurfaces represent a rapidly evolving field, fundamental research remains essential and highly impactful. A critical component of any metasurface is the dielectric substrate on which it is fabricated – a factor that holds significance across all spectral ranges. This work focuses specifically on metallic terahertz metasurfaces operating in transmission mode. It provides an overview of various substrates (Ge, Si, SiO₂, TPX) and discusses design principles for such metasurfaces, including magnetic and electric dipole surface plasmon resonances, Fano resonances, and quasi-bound states in the continuum. We initially simulated the structures using COMSOL Multiphysics software and then confirmed the results experimentally by detecting various concentrations of bovine serum albumin. Our study systematically examines how real-metal modeling and substrate selection influence the Q-factor and sensing performance, in contrast to earlier research that either fixed the substrate type or modeled the metal as a perfect electric conductor. This dual approach provides valuable guidance for designing high-Q, low-loss terahertz metasurface biosensors. Our results demonstrate that for all types of terahertz metasurfaces operating in transmission mode, using a low-refractive-index substrate enhances sensor sensitivity, making it the preferred choice for sensing applications. This opens new prospects for the design of high-sensitivity biosensors.

## Introduction

Sensors based on terahertz metasurfaces (THz MSs) are starting to gain significant attention in the sensor community^1^. The main focus is on biosensing due to the unique properties of THz radiation. It is non-ionizing (safe for biological samples and operators^2^, sensitive to water content, and capable of detecting weak molecular interactions, as resonances of hydrogen and van der Waals bonds fall within the THz spectral range^3,4^. Additionally, rotational and vibrational modes of big molecules also fall within this range. THz-based biosensors have shown promise for label-free, non-invasive detection of biomolecules^5–7^. However, potential applications are not limited to biosensing. For example, THz MSs can be utilized for controlling wave propagation^8–11^, THz modulation for high-speed communication^12,13^, spectroscopy^14^, imaging^15^, and beam scanning^16^, as the demand for smart, integrated, and multifunctional devices grows^17,18^.

The THz MS typically consists of a periodic metal pattern deposited on a dielectric material that is called substrate. These metasurfaces address a major challenge in THz biosensing: the stark difference between the THz wavelength (on the order of hundreds of micrometers) and the size of biological objects, such as cells, bacteria, viruses, proteins, and DNA (typically less than a micrometer)^19^. This is because the gap size in the metal pattern, which typically measures around one micrometer, determines the properties of the THz MS^20^.

The substrate is a critical component of any THz MS, as its properties significantly influence sensor’s sensitivity. The thickness, refractive index, and material losses are key factors to consider. Because sensors typically operate in transmission mode, low-loss materials are preferred, such as high-resistivity silicon and germanium or low-loss plastics like PE, PTFE, and TPX^21^. Studies have shown that reducing substrate thickness increases sensitivity, as thicker, high-index materials add capacitance to the resonator, masking spectral changes caused by the analyte^22,23^. Similarly, using lower refractive index materials is recommended for enhancing detection sensitivity^24^. However, comprehensive research on the influence of substrate refractive index is still lacking.

The choice of substrate is crucial for the performance, sensitivity, and overall effectiveness of THz biosensors. Substrate materials can be broadly categorized into high-index and low-index materials. High-index substrates, such as silicon and germanium, suffer from significant reflection losses due to impedance mismatch with the air. This reduces the interaction with target biomolecules, lowering sensitivity. These materials also exhibit high dielectric and absorption losses, causing wave attenuation and energy dissipation as heat, which further diminishes sensing efficiency. In contrast, low-index substrates like quartz (*n* = 2.1) and TPX (*n* = 1.46) exhibit much lower reflection losses, allowing for more efficient THz wave transmission, stronger interactions with biomolecules, and higher sensitivity. This makes them ideal for THz biosensing applications. Our work aims to verify these assumptions.

Through plasmonic^25^ and electric dipole interactions^26^, Fano^27,28^ and quasi-bound states in the continuum resonances^29,30^ have been extensively studied in the visible and near-infrared regimes, providing valuable insights for designing high-Q resonances. Building on these concepts, our research explores similar phenomena in the THz range, specifically tailored for substrate-sensitive metamaterial detection. Sensors using THz MSs operate in transmission (or reflection) mode, where the transmission (or reflection) spectrum of THz radiation is measured after broadband THz illumination^24^. The THz MS typically produces a resonance in the spectrum, often visible as a dip in transmission (or reflection)^20^ described by the Lorentzian curve. Another well-known optical resonance is the Fano resonance, which arises when a discrete localized state interacts with a continuum of states. Before Fano’s conceptual work on resonance in 1961^31^, the Lorentzian formula was widely regarded as the fundamental line shape for resonances. Unlike Lorentzian resonance, Fano resonance exhibits a characteristically asymmetric line shape. This asymmetry arises from the constructive and destructive interference between a broad spectral line (or continuum) and a narrow discrete resonance^32^. In recent years, Fano resonances have been extensively utilized as a promising resonant mechanism for biosensing applications in various THz metamaterial structures^33–35^. Thanks to their narrow bandwidth, strongly asymmetric line shape, and high quality (Q) factor, Fano resonances in metasurfaces significantly enhance local optical near-fields and promote light-matter interactions at specific frequencies within subwavelength resonators.

Furthermore, generating ultrahigh-Q resonances, which enhance light confinement and reduce leakage, has been closely linked to bound states in the continuum (BICs)^36^. These are localized waves that defy conventional expectations by remaining confined despite coexisting with radiative spectra. Initially proposed in quantum mechanics, BICs have been identified across various wave systems and materials, such as photonic crystals, optical waveguides, and graphene. While ideal BICs with infinite lifetimes are unattainable, quasi-BICs (QBICs) achieved through induced perturbations offer ultrahigh-Q resonances and significant near-field enhancement, enabling precise control over light-matter interactions^37,38^. The recent work by R. Wang et al.^39^ on a high-Q (QBIC) THz biometasensor, featuring split-ring resonators on a 5 mm TPX substrate functionalized with AuNP-antibody conjugates and achieving an impressive sensitivity of 674 GHz/RIU, has inspired us to investigate the impact of substrate properties on the sensitivity of THz biometasensor. In biosensors, the attachment of biological matter to the THz MS causes a shift in the resonance frequency (*f*_*0*_), which serves as the sensor’s response. The operation of biosensors is typically simulated by covering the THz MS with a layer of analyte of a specified thickness and refractive index. Previous studies have explored the effect of analyte layer thickness^40^.

Unlike earlier works that are mostly interested in unit cell shape, plasmon hybridization, or material tunability (such as graphene or spiral-based structures)^41–44^, this work addresses a major but understudied element of THz biosensor design: the substrate. We show, by modelling and experimental validation, that the substrate refractive index greatly affects evanescent field confinement, effective mode index modulation, and hence sensor sensitivity. Furthermore underlining the need of precise material modelling in future biosensors, we show that representing metals as ideal conductors results in an overestimation of Q-factors in high-performance devices. This study investigates also substrate choice impact on different design of THz metasurfaces, including magnetic and electric dipole surface plasmon resonances, Fano resonances, and QBIC. Standard split ring resonator (SRR) structure produces simple electric or magnetic dipole moment^45–48^. The Fano resonance structure consists of a continuous metallic wire coupled with a split-ring resonator (SRR) and is characterized by a sharp, asymmetric spectral profile^49^. This asymmetry arises from the coupling between a discrete resonance mode (SRR) and a broad continuum mode (continuous wire), resulting in enhanced electromagnetic field confinement and reduced radiative losses. These properties make the structure a promising candidate for biosensing applications. On the other hand, the cross structure^39^ exploits symmetry-breaking mechanisms to achieve high-Q resonances. A BIC is a resonance state that remains localized and does not radiate energy. By introducing a slight perturbation to the metasurface design, it is possible to control the leakage of electromagnetic energy, leading to strong electromagnetic energy confinement with minimal radiation losses. The cross structure achieves high-Q resonances with exceptionally low radiation losses, which is critical for detecting biomolecules. Additionally, the cross structure offers a much higher Q-factor than the Fano resonance structure, significantly enhancing the sensitivity of the metasensor. The asymmetric split-ring resonator (ASRR) structure is another promising candidate for achieving high Q-factors by substantially reducing radiation losses^50^. The ASRR can excite two destructively interfering bright modes in its two unequal sections. This concept, proposed by^51^, requires the two sections of the ASRR to be similar but not identical, which imposes high fabrication demands.

## Simulations

In the simulation, X-polarized (or Y-polarized) light propagates along the Z-axis and is incident perpendicularly onto the metasurface. To determine the electromagnetic response of our proposed metasurface, including the transmission amplitude, we used a single unit cell geometry to perform full-wave electromagnetic simulations. We employed the finite element method (FEM) using COMSOL Multiphysics (version 6.1) with the RF module to simulate the metallic THz metasurfaces. Perfectly matched layers (PMLs) were used to truncate the infinite space along the Z-axis, while periodic boundary conditions were applied along the X and Y axes to enable realistic simulations. A fine, triangular mesh tailored for metamaterial structures was used to discretize the subdomains of the proposed design in the COMSOL simulations, achieving a balance between high accuracy and computational efficiency. Since the typical conductivity of metals in the THz region is approximately 10⁷ S/m, the metal was modeled as a perfect electric conductor (PEC) throughout the simulation if not mentioned otherwise. A metal layer thickness of 110 nm was used.

**Fig. 1:**
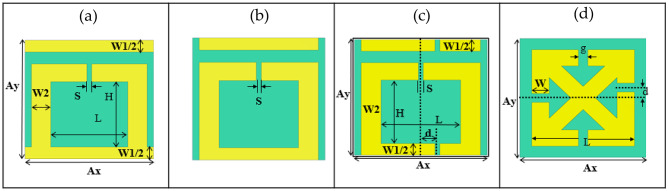
Schematics of unit cell of the structures studied: (**a**) Fano resonance structure^49^; (**b**) purely SRR structure; (**c**) asymmetrical SRR (ASRR) structure. The Fano resonance structures (**a**) are characterized by specific dimensions: both Ax and Ay measure 40 μm, W_1_ is 8 μm, W_2_ is 6 μm, L is 24 μm, H is 22 μm, and the gap width is denoted as S = 1.5 μm. The SRR (**b**) and ASRR (**c**) structures have the same dimensions. Just in ASRR (**c**) there is an additional gap of 1.5 μm shifted by the distance d = 5 μm; (**d**) cross structure^39^ has the dimension W = 11 μm, L = 65 μm, Ax = Ay = 80 μm, d = 10 μm and g = 6 μm.

We analyzed four different designs of metallic THz metasurfaces to study the influence of a substrate on the sensitivity. Figure 1 illustrates the unit cells of the designs studied. The first design involves a modified SRR together with a transmission line exhibiting Fano-type resonance (Fig. 1a) taken from Zhaofang Li et al.^49^. Where Fano resonance in terahertz metamaterials arise from integration of split-ring resonators with continuous wire structures on the quartz substrate. This design was not yet investigated for experimental applications involving biomolecules. The second design is based on a standard SRR (Fig. 1b) with dimensions consistent with Fano resonance structure. The third design is an ASRR with a second gap that demonstrates the QBIC response (Fig. 1c). To address this, we modified the SRR structure into the ASRR structure by introducing an asymmetry parameter, *d*, while keeping the geometric parameters consistent with the Fano resonance structure. Finally, the fourth design features a cross-shaped structure (Fig. 1d), which replicates a design from Ref^39^. to further validate our simulation results. This design is particularly notable as it is fabricated on a TPX substrate, which is uncommon for biosensor applications. Another interesting feature of this design is its magnetic dipole-type of the QBIC excitation.

For the substrate, we selected four materials with different refractive indices: TPX (*n* = 1.46)^21^, quartz (SiO₂, *n* = 2.1), silicon (*n* = 3.36), and germanium (*n* = 4)^52^. All substrates were surrounded by air (*n* = 1), and their thickness was set to 280 μm. For the sensitivity analysis, the analyte was modeled as a fixed layer with a thickness of 10 μm, and its refractive index (n) was varied from 1 to 2 to simulate the coverage of the metasurface with biological matter^40^. This range is representative of biomaterials, such as proteins, which typically have refractive indices between 1 and 2. We selected a single analyte layer thickness for this study, as previous research (see Ref.^40^. has shown that frequency shifts saturate at around 5 μm of analyte layer thickness.

For the Fano resonance structure (Fig. 1a), the incident electromagnetic wave is polarized in the X-direction along the continuous gold wires. The unit cell of the Fano resonance structure, depicted in Fig. 1a, includes dimensions detailed in the figure caption. Essentially, this configuration combines the SRR structure (Fig. 1b) with a transmission line. Similarly, for the SRR structure (Fig. 1b), the incident electromagnetic wave is also polarized in the X-direction. However, for the ASRR and cross structures (Fig. 1c and d, respectively), the incident electromagnetic wave is polarized in the Y-direction.

## Experimentals

**Fig. 2:**
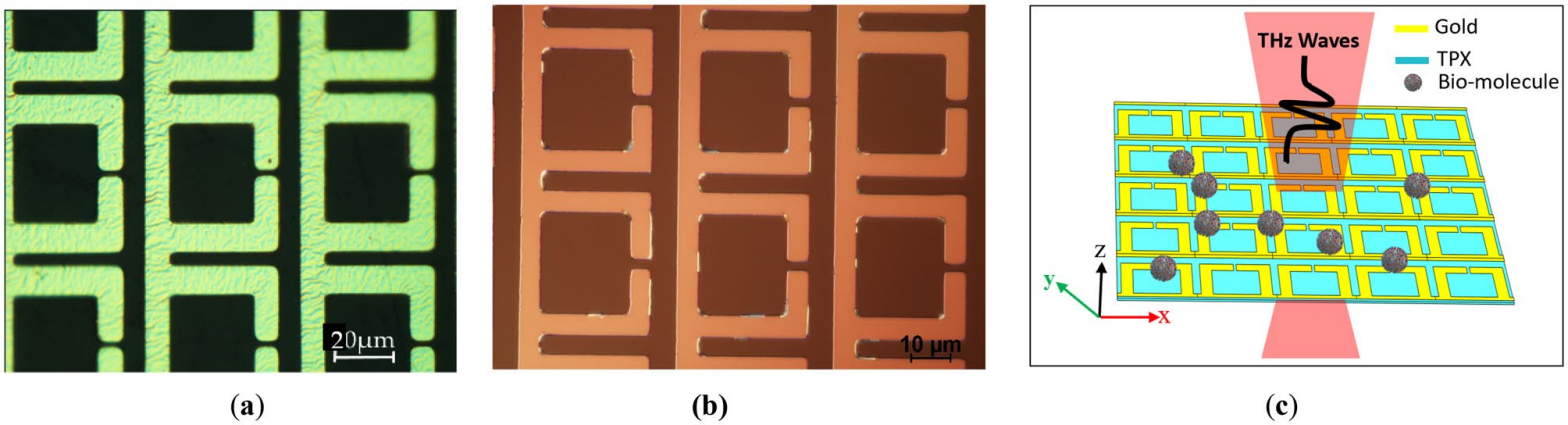
Optical photographs of the Fano resonance structures with the gap of 1.5 μm fabricated on two different substrates: (**a**) TPX (in Namarski contrast) and (**b**) silicon. (**c**) The schematics of the metallic planar Fano structure on a TPX substrate.

To compare simulations with experiment we processed THz MS on TPX (Fig. 2a) and on silicon (Fig. 2b). The metasurface we chose for fabrication was the structure displaying the Fano resonance (Fig. 1a).

The proposed Fano structure taken for fabrication is composed of SRRs connected by periodic metallic wires. It is design to assist Fano resonances while avoiding higher-order resonances and dark modes. In contrast to traditional designs, the Fano resonance in this case is caused by interference between the SRR’s bright resonant mode and the wires broadband transmission continuum. We fabricated the Fano resonance structure on two different substrates: a 500 μm thick TPX substrate (Fig. 2a) and a 280 μm thick high-resistance silicon substrate (Fig. 2b). The metasurface was created using laser lithography, followed by e-beam evaporation sputtering to deposit a 100 nm gold (Au) layer with a 10 nm titanium (Ti) adhesion layer. The lift-off process resulted in a metasurface array measuring 4 mm × 4 mm. Experimental data were collected using Toptica’s TeraFlash Pro Time Domain Spectroscopy (TDS) system. The time profile of the THz electric field was measured by varying the time delay between the probe and the THz beam within a 200 ps range, corresponding to a frequency resolution of 5 GHz. The frequency profile of the THz electric field was then obtained by applying the Fourier transform to the time-domain data. The TDS measurements were conducted in transmission mode, with the THz beam focused on the MS array. All the measurements were performed in a nitrogen-purged environment to eliminate interference from water vapor absorption lines. During data analysis, the time trace was truncated just before the first reflection peak to prevent oscillations in the frequency spectrum caused by multiple reflections within the sample.

To experimentally simulate the effect of covering the metasurface with an analyte of varying refractive indices, we examined the resonance position in THz transmission as a function of concentration of bovine serum albumin (BSA) protein deposited on the metasurface. This approach mimics the effect of increasing the refractive index, as in the simulations. The observed shift (Δf) is expected to saturate at the highest BSA concentration, corresponding to 0.8 RIU, given that the reported refractive index of BSA is 1.8^53^. For the experiment, we prepared BSA solutions with concentrations ranging from 1 to 40 mg/ml. Initially, we measured the clean metasurfaces using TDS. We then deposited different BSA concentrations onto separate metasurface chips, allowed them to dry under ambient conditions, and remeasured them to record the resonance shift for each concentration. These experiments were performed for Fano resonance structure on silicon and TPX substrates.

## Results and discussion

**Fig. 3:**
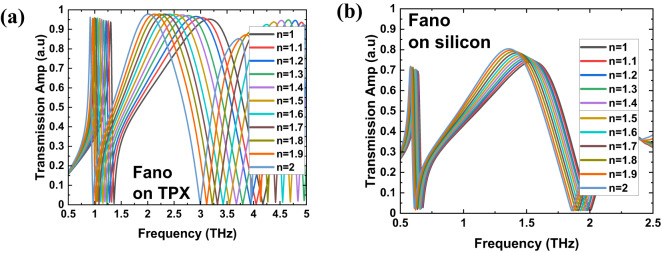
The results of simulations of the transmission with 10 μm layer of dielectric of a given refractive index for THz MS displaying Fano resonance (Fig. 1a) for two kind of substrates: TPX (**a**) and silicon (**b**).

**Fig. 4:**
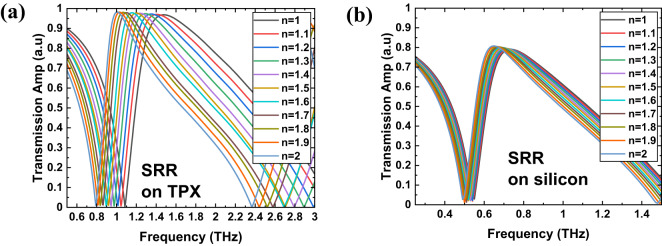
The results of simulations of the transmission with 10 μm layer of dielectric of a given refractive index for THz MS with SRR design (Fig. 1b) for two kind of substrates: TPX (**a**) and silicon (**b**).

**Fig. 5:**
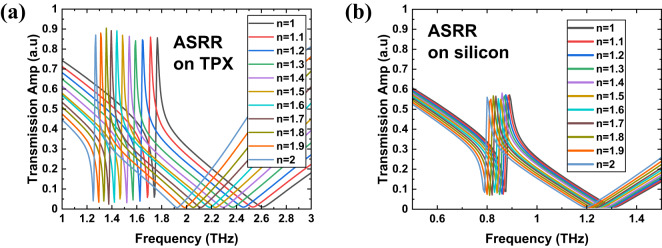
The results of simulations of the transmission with 10 μm layer of dielectric of a given refractive index for THz MS with ASRR design (Fig. 1c) for two kind of substrates: TPX (**a**) and silicon (**b**).

**Fig. 6:**
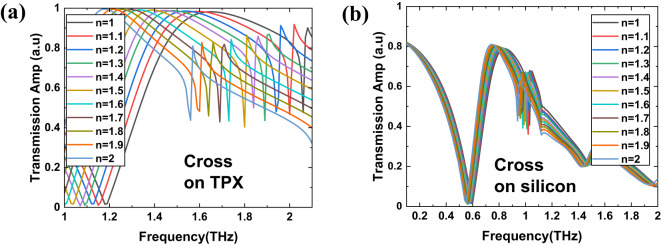
The results of simulations of the transmission with 10 μm layer of dielectric of a given refractive index for THz MS with cross design (Fig. 1d) for two kind of substrates: TPX (**a**) and silicon (**b**).

For all the structures from Fig. 1, and for four types of substrates, we conducted simulations to explore their sensing capabilities for biosensing applications by observing how the transmission spectra vary with changes in the refractive index (n) of the covering material. Figures 3, 4, 5 and 6 present the simulation results of the transmission spectra for various design principles: Fano resonance, SRR, ASRR, and cross structures, respectively, on two types of substrates: TPX (a) and silicon (b). The results reveal a series of asymmetric line shapes, which are the primary characteristic of the Fano resonance, ASRR, and cross structures, whereas the pure SRR structure exhibits a typical Lorentzian line shape.

**Fig. 7:**
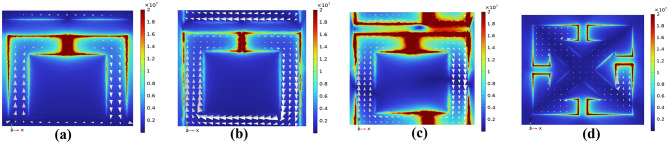
The results of simulations of the electric field intensity and current distribution for all designs studied: (**a**) Fano resonance structure, (**b**) SRR structure, (**c**) ASRR structure, (**d**) cross structure.

To understand the origin of the transmission peak for each design, we analyzed the current distributions within the structures based on simulation results (Fig. 7). In the Fano resonance structure (Fig. 7a), the currents in the overlapping region of the split-ring resonators and continuous wires flow in the opposite direction to those in the rest of the Fano structure, leading to destructive interference and resulting in the characteristic Fano-type transmission peak (more details can be found in^49^. The electromagnetic field is highly confined within the split gaps of the Fano resonator, making metal-based Fano resonator platforms well-suited for exploring light–matter interactions at terahertz frequencies. In the case of the asymmetric split-ring resonator (ASRR), when excited with a Y-polarized THz wave at d = 5 μm, strong surface currents are induced. These currents are redistributed in terms of phase and magnitude, indicating that the bound state is coupled to the incident radiation, thereby giving rise to a quasi-bound state in the continuum (QBIC) resonance (more details can be found in^39^.

Figure 1c and d illustrate the mirror symmetry of the ASRR and cross-shaped structures, respectively. The ASRR structure exhibits mirror symmetry in the YZ-plane along a central vertical axis, while the cross structure shows mirror symmetry in the XZplane along a central horizontal axis. In the ASRR, asymmetry is introduced by shifting the gap by 5 μm relative to the vertical symmetry line. Similarly, in the cross-structure, the gap is displaced by 10 μm relative to the horizontal symmetry line. As shown in Figs. 5 and 6, the excitation of a trapped-mode resonance is evident in the transmission spectra, regardless of the substrate used, when the structures are illuminated with an Y-polarized THz wave. A strong current (see Fig. 7c,d) is observed along the right arm of the ASRR and the cross-structure, indicating strong coupling with the incident radiation and significant energy leakage into the continuum. These trapped modes are typically forbidden by the inherent symmetry of the metamaterial unit cell. However, by breaking the structural symmetry, a sharp and narrow Fano resonance emerges, characterized by its distinctive asymmetric line shape. This analysis supports our simulations results and show that they are correct.

As we can observe in Figs. 3, 4, 5 and 6, there is a redshift in the frequency dip as we transition from *n* = 1 to *n* = 2. It can be seen that the frequency shift per unit refractive index decreases as we move from a substrate with lower relative permittivity (TPX) to one with higher relative permittivity (silicon). This behavior is due to two primary factors: (i) the increase in non-radiative losses associated with substrates of higher permittivity, and (ii) the enhancement of electromagnetic field confinement within the substrate, rather than extending into the surrounding medium where the analyte is present. This confinement reduces the overlap between the evanescent fields of the metasurface and the analyte layer, decreasing the interaction volume and, consequently, the sensor’s sensitivity^54,55^. Additionally, there is a notable drop in transmission amplitude for silicon and germanium substrates, driven by elevated reflection losses when moving from low- to high-refractive-index materials (reflection at the interface of two materials with a large difference in refractive indices).

**Fig. 8:**
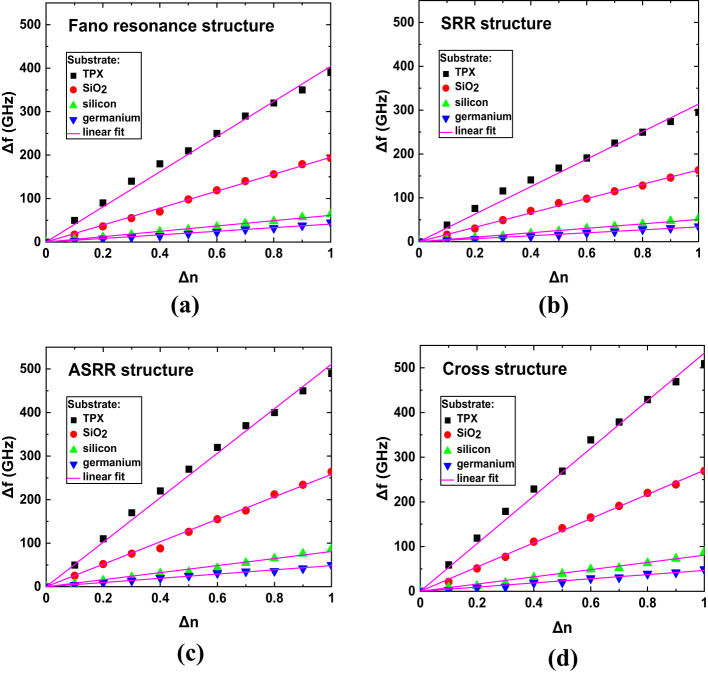
The shift in frequency of resonance peak (Δf) as a function of refractive index change (Δn) for various substrates, for various structures: (**a**) Fano resonance, (**b**) SRR, (**c**) ASRR and (**d**) cross. Lines depicts linear fits for simulation results (points).

From the simulation we also extracted refractive index sensitivity, which can be calculated as Δf/Δn for various substrates. The graphs in Fig. 8 show a linear correlation between the frequency shift (Δf) and the refractive index change (Δn). Notably, the sensitivity decreases when moving from TPX (black squares) to germanium (blue inverted triangles). This trend holds for all the structures studied. From the linear fit to the simulation data (points) we obtained sensitivity values (in GHz/RIU units), which are summarized in Table 1.

Strong coupling effects, such as hybridized surface plasmon resonances and cavity-enhanced modes, have made visible and near-infrared plasmonic resonators and metal–insulator–metal waveguide structures highly sensitive and capable of strong field confinement. For example, studies using bowtie-shaped cavities^56^ and embedded baffles have demonstrated that multipolar interference and tailored field localization can significantly enhance the Q-factor and refractive index sensitivity^57^. Although these designs operate only at shorter wavelengths, the underlying principles of mode confinement and light–matter interaction remain highly valuable. Building on these concepts, our THz-based metasurface sensor incorporates realistic material modeling and substrate engineering to enhance detection sensitivity through similar field localization strategies. At resonance frequencies, metamaterials (MMs) can be regarded as resonant cavities that efficiently store substantial electromagnetic (EM) energy within subwavelength-scale volumes^54,58^. The sensitivity of THz metasurface biosensors is directly related to the degree of interaction between the analyte and the localized resonant EM fields. This relationship is quantitatively described by dielectric perturbation theory, which relates the relative resonance frequency shift (Δf/f₀) to the permittivity perturbation (Δε) and the spatial distribution of the electric and magnetic fields within the resonator. The following provides the general expression:

1 $$\:\frac{\varDelta\:f}{{f}_{0}}\:=\:-\:\frac{{\int\:}_{\varDelta\:\varvec{V}}\:\varDelta\:\varvec{\epsilon\:}{\:\left|\overrightarrow{{\varvec{E}}_{0}}\right|}^{2}\varvec{d}\varvec{V}}{{\int\:}_{\varvec{V}}\:\varvec{\epsilon\:}{\:\left|\overrightarrow{{\varvec{E}}_{0}}\right|}^{2}\varvec{d}\varvec{V}+{\int\:}_{\varvec{V}}\:\varvec{\mu\:}{\:\left|\overrightarrow{{\varvec{H}}_{0}}\right|}^{2}\varvec{d}\varvec{V}}$$

where Δε is the change in permittivity caused by the analyte, E₀ and H₀ are the unperturbed electric and magnetic fields, V is the resonator volume, and ΔV is the analyte volume. The above equation demonstrates that the overlap between the analyte volume ΔV and the local electric field energy density ∣E_0_∣^2^ is directly proportional to the resonance shift. This can be reduced to the following when thin-film analytes and dominant electric-field interaction are involved:

2 $$\:\frac{\varDelta\:f}{{f}_{0}}\:\approx\:\:-\:\frac{\varDelta\:\varvec{\epsilon\:}\varvec{d}\varvec{A}}{2{\varvec{\epsilon\:}}_{\varvec{s}\:{\varvec{V}}_{\varvec{e}\varvec{f}\varvec{f}}}}$$

Here, d and A represent the thickness and area of the analyte layer, ε_s_ is the substrate permittivity, and V_eff_ is the resonant structure’s effective mode volume. This simplified form shows that by increasing field confinement into the analyte area, sensitivity increases in substrates with smaller dielectric constants (ε_s_). The overlap between the analyte and the high-field area can be increased with a lower-index substrate thanks to stronger field asymmetry and evanescent field penetration into the sensing volume. Therefore, improving the substrate’s properties is crucial to increasing the frequency shift and sensitivity of THz metasurface biosensors.

We also calculated the Q factor for all the structures and substrates simulated, and the values are provided in Table 2. The Fano resonance structure has an advantage in terms of the Q factor over the SRR structure, due to the fact that Fano resonance arises from the interference between the broad continuum mode (radiative mode) and the discrete state (dark mode). This interference effectively cancels out the radiation, trapping the energy in the narrow mode. These narrow modes couple weakly to free-space radiation, resulting in an increased Q factor. However, the SRR is an open, radiative structure that primarily arises from the oscillation of electric and magnetic dipoles. These modes strongly couple to free-space radiation and readily emit energy. The high level of radiation losses leads to a poor Q factor. The sensitivity of the metasensor depends on the ability of the metamaterial to strongly confine the electromagnetic field at the location where the analyte is present. A high Q factor (by suppressing radiative and non-radiative losses) ensures a narrow resonance peak for the accurate detection of the resonance shift and maintains a small mode volume^58^.

**Table 1:** Sensitivities (in GHz/RIU units) from the simulations for various substrates and design schemes of THz MS.

Substrate	Design			
	Fano resonance	SRR	ASRR	cross
TPX	404	314	511	533
SiO_2_	195	164	258	271
Si	62	51	81	81
Ge	41	34	48	47

**Table 2:** Quality factor from the simulations for various substrates and design schemes of THz MS.

Substrate	Design			
	Fano resonance	SRR	ASRR	cross
TPX	55	5.9	192	286
SiO_2_	60	5.8	180	254
Si	51	5.6	105	224
Ge	57	5.9	128	155

**Fig. 9:**
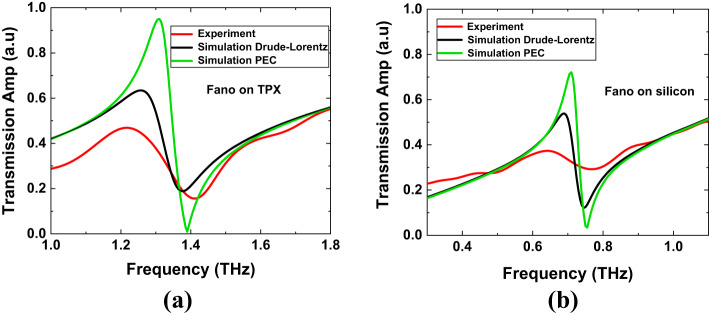
The comparison of the experimental results (red line) with the simulation (*n* = 1) for two models of metal conductivity: the Drude-Lorentz model and the PEC model. The comparison is shown for the Fano resonance structures (all with W_1_ = 6 μm) featuring a gap of 1.5 μm (as in Fig. 2), fabricated on two different substrates: (**a**) TPX (see Fig. 2a) and (**b**) silicon (see Fig. 2b).

Figure 9 shows the transmission spectra of the experimental results compared to the simulation (*n* = 1) results for two different models of metal conductivity: the Drude-Lorentz model and the PEC model. All for the Fano resonance structure on TPX and silicon with a 1.5 μm gap, same as depicted in Fig. 2. We see a good agreement with the position of the Fano resonance. Since TPX is a lossless material, much of the electromagnetic energy is confined in the metasurface rather than interacting with the substrate. From both the experimental and simulation results, we can clearly see that the transmitted signal from TPX is much sharper than that from silicon. When considering the metasurface as a PEC, there will be large confinement of the electric field in the capacitive gap, i.e., the transmitted amplitude of the Fano resonance will be higher in the simulation compared to the experimental data. Indeed, in^50^, the authors show that the assumption of PEC becomes highly inaccurate for high-Q asymmetric Fano resonant metamaterial structures. They found that the resonance behavior is extremely sensitive to the conducting properties of the metal from which the resonators are made. Therefore, we also present the results of simulations using the Drude-Lorentz model for metal conductivity. The dielectric constant of metal (gold) is described by the Drude model $\:\epsilon\:=1-\frac{{\omega\:}_{p}^{2}}{{(\omega\:}^{2}+i\gamma\:\omega\:)}$ in which we take the plasma frequency $\:\frac{{\omega\:}_{p}}{2\pi\:}$ = 2175 THz and the collision frequency $\:\frac{\gamma\:}{2\pi\:}\:$= 9 THz. It is evident that this model shows better agreement with the experimental curve compared to the PEC model. However, discrepancies especially in the amplitudes remain, which can be attributed to imperfections in the fabrication of the MS, particularly in the gap region.

Zhaofeng Li et al.^49^ has studied the Fano resonance structure, demonstrating how Fano resonance in THz metamaterials can be tuned and analyzed for potential THz sensing applications, though the focus is not specifically on biosensing applications. In our simulation, we focus more on the possibilities of using the Fano resonance structure specifically for biosensing applications. In the first step toward this, we simulated the Fano resonance structure on various substrates and calculated the sensitivity for each substrate. Based on our simulation results, we found that the Fano resonance structure indeed shows much higher sensitivity for lower-index substrates and decreases as we move toward higher-index substrates. This makes the Fano resonance structure a potential candidate for biosensing applications. Meanwhile, R. Wang et al.^39^ studied a cross structure fabricated on a 5 mm thick TPX substrate, specifically focusing on biosensing applications using the QBIC mechanism. We also simulated the cross structure, specifically focused on biosensing applications, but for various substrates. From our simulations, we found that the QBIC mechanism offers a much higher Q-factor when using the cross structure on a TPX substrate, resulting in much higher sensitivity.

For the Fano resonance structure, our simulations closely match the results from the respective article, proving the validity of our studies. On the other hand, our simulated sensitivity for the cross structure is 533 GHz/RIU and is considerably less than what was claimed in^39^ namely 674 GHz/RIU. Our results were confirmed experimentally, what is described in the paragraphs below, and can be trusted more. Whereas in Ref. 39 authors presented only the result of simulation. The reason behind this error likely comes from fundamental differences between FDTD (used in Ref^39^. and FEM methods (our simulations), especially when high Q-factor designs are analyzed. The FEM method calculates steady state results, where FDTD algorithm reproduces a natural process of wave propagation and convergence to the steady state depends on the Q-factor of the modeled structure^59^. FDTD suffers from numerical dispersion, staircasing errors, and long simulation times when modeling high-Q resonators, leading to frequency shifts and artificial Q-factor reduction^60^. The FEM method, on the other hand, provides higher frequency accuracy through adaptive meshing and eigenmode solvers, making it superior for curved geometries and complex materials^61^.

Our simulation results show that as we move from lower-index to higher-index substrates, the sensitivity decreases drastically. Variations in the substrate’s refractive index can significantly alter the coupling efficiency between the metasurface and free-space radiation. High-index substrates can introduce additional phase shifts and modify the effective optical path lengths, which can affect the resonance conditions of the metasurface. With a higher refractive index substrate, the electromagnetic fields tend to be more confined within the substrate rather than extending into the surrounding medium, where the analyte is present. This confinement reduces the overlap between the evanescent fields of the metasurface and the analyte layer, decreasing the interaction volume and, consequently, the sensor’s sensitivity. In THz metasurfaces, most of the energy (electric and magnetic) is confined in the vicinity of the metasurface. Changing the refractive index of the thin dielectric layer alters the distribution of the electromagnetic field around the metasurface. This changes the effective permeability and permittivity around the metasurface, leading to the redshift of the resonance peak^54,58^. The biosensing mechanism based on changes in refractive index works better when the resonance frequency varies with respect to refractive index changes, as this forms the basis for calculating sensor sensitivity to biomolecules. Greater frequency variation is observed with TPX, while it is less with silicon. This is why the silicon spectrum appears stable, which is not favorable in terms of sensor sensitivity.

**Fig. 10:**
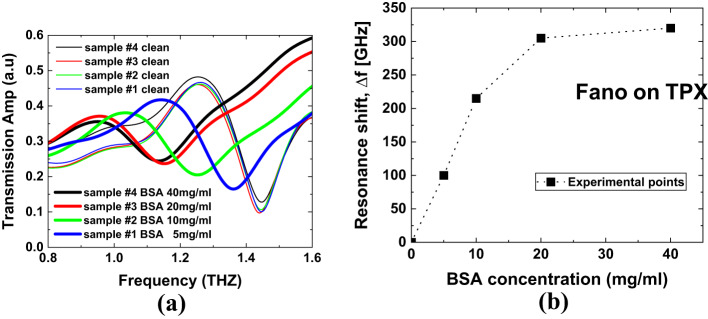
(**a**) Transmission spectra for four samples on TPX with varying BSA concentrations. (**b**) Fano resonance shift as a function of BSA concentration, derived from the data in panel (**a**). The errors in panel (**b**) from fitting the data in panel (**a**) do not exceed 5 GHz (our TDS resolution) and are smaller than the size of the squares marking the experimental points.

To confirm the quality of the metal pattern, optical microscopy was sufficient, as the metasurface features are on the micrometer scale. To verify fabrication consistency, we used THz-TDS to examine the transmission spectra of four nominally identical 4 mm × 4 mm samples (see Fig. 10a – thin colored lines). The minimal variation in resonance peaks among the samples indicates high fabrication quality and uniformity. The final validation of our method and numerical results is presented in Fig. 10, where we show the Fano resonance shift (Fig. 10b) as a function of BSA protein concentration, derived from the transmission spectra (Fig. 10a). This experiment effectively simulates the process of covering the metasurface with a material of increasing refractive index, consistent with our simulations. The observed resonance shift (Δf) reaches saturation at 320 GHz for the highest tested BSA concentration of 40 mg/ml. This saturation occurs because, as the analyte thickness increases, the upper portion of the analyte gradually approaches or even exceeds the spatial extent of the bound electromagnetic field. As a result, the portion of the analyte outside the field contributes less to the frequency shift, leading to saturation. This behavior aligns with simulation results for a refractive index increase of 0.8, corresponding to a final refractive index of 1.8 (see Fig. 7a, Δn = 0.8). Notably, this value (*n* = 1.8) is in excellent agreement with previously reported refractive index measurements for BSA in the literature^53^. Additionally, the experimentally measured sensitivity for silicon, calculated as described above, is 74 GHz/RIU, which closely matches the simulation result of 62 GHz/RIU.

## Conclusions

In conclusion, we primarily conducted a simulation study on the effect of various substrates on THz metasurfaces (operating in transmission mode) and their sensitivity – a crucial parameter for THz-based biosensors. Our simulations were validated experimentally and also compared with previous experimental studies. To this end, we experimentally investigated the THz transmission spectra of the Fano resonance structure on TPX and silicon substrates, including measurements for various concentrations of BSA. These findings further confirm the accuracy and reliability of our approach in detecting refractive index changes using THz spectroscopy. The strong correlation between the experimental and simulated data highlights the potential of our metasurface-based sensing platform for precise biochemical analysis. In future work, we aim to extend this technique by integrating microfluidic pathways and metasurfaces for label-free, real-time biosensing. Enabling the detection of additional biomolecules will significantly enhance the utility of this approach. Moreover, potential applications in environmental monitoring and biomedical diagnostics may emerge. Our study demonstrates that designing metasurfaces – especially those exhibiting Fano resonance and quasi-bound states in the continuum (QBIC) – on low-refractive-index substrates results in higher sensitivity compared to those on high-index substrates. We also investigated the effect of transmission amplitude in the Fano resonance structure when modeling the metasurface as a perfect electric conductor (PEC). Our experiments revealed that conductivity is a critical parameter in designing high-Q metamaterials. These results can be leveraged to design high-sensitivity, THz-based biosensors. Overall, our findings will contribute to the design and fabrication of high-Q metasurfaces, paving the way for more efficient and easier-to-process sensing devices.

## Data Availability

The raw data supporting the conclusions of this article will be made available by the authors on request. Please contact the corresponding author at sakowicz@unipress.waw.pl .
